# Accurate semi-supervised automatic speech recognition for ordinary and characterized speeches via multi-hypotheses-based curriculum learning

**DOI:** 10.1371/journal.pone.0333915

**Published:** 2025-10-21

**Authors:** Ka Hyun Park, Junghun Kim, U Kang

**Affiliations:** 1 Department of CSE, Seoul National University, Seoul, Republic of Korea; 2 IPAI, Seoul National University, Seoul, Republic of Korea; VIT Bhopal University, INDIA

## Abstract

*How can we build accurate transcription models for both ordinary speech and characterized speech in a semi-supervised setting?* ASR (Automatic Speech Recognition) systems are widely used in various real-world applications, including translation systems and transcription services. ASR models are tailored to serve one of two types of speeches: 1) ordinary speech (e.g., speeches from the general population) and 2) characterized speech (e.g., speeches from speakers with special traits, such as certain nationalities or speech disorders). Recently, the limited availability of labeled speech data and the high cost of manual labeling have drawn significant attention to the development of semi-supervised ASR systems. Previous semi-supervised ASR models employ a pseudo-labeling scheme to incorporate unlabeled examples during training. However, these methods rely heavily on pseudo labels during training and are therefore highly sensitive to the quality of pseudo labels. The issue of low-quality pseudo labels is particularly pronounced for characterized speech, due to the limited availability of data specific to a certain trait. This scarcity hinders the initial ASR model’s ability to effectively capture the unique characteristics of characterized speech, resulting in inaccurate pseudo labels. In this paper, we propose a framework for training accurate ASR models for both ordinary and characterized speeches in a semi-supervised setting. Specifically, we propose MOCA (Multi-hypotheses-based Curriculum learning for semi-supervised Asr) for ordinary speech and MOCA-S for characterized speech. MOCA and MOCA-S generate multiple hypotheses for each speech instance to reduce the heavy reliance on potentially inaccurate pseudo labels. Moreover, MOCA-S for characterized speech effectively supplements the limited trait-specific speech data by exploiting speeches of the other traits. Specifically, MOCA-S adjusts the number of pseudo labels based on the relevance to the target trait. Extensive experiments on real-world speech datasets show that MOCA and MOCA-S significantly improve the accuracy of previous ASR models.

## Introduction

*How can we train an accurate transcription model for speech of the general population and those of individuals with specific traits in a semi-supervised setting?* Speech is a primary and essential means of human communication. Developing accurate automatic speech recognition (ASR) systems is essential to efficiently leverage the growing volume of speech data for various applications, including voice search [1,2], speech command recognition [3], automatic transcription of spoken content [4,5], information extraction [6,7], and machine translation [8,9].

Previous ASR models are categorized to address two types of speech: 1) *ordinary speech* from the general population, and 2) *characterized speech* from speakers with specific traits such as accents or speech disorders. ASR models for ordinary speech generate transcriptions without accounting for specific traits including gender or age [10]. On the other hand, ASR models for characterized speech are designed to transcribe speech from individuals with specific characteristics, such as regional accents or speech impairments [11–13].

The limited availability of labeled speech data and the high cost of manual annotation have recently driven increased attention toward advancing semi-supervised ASR approaches [14–16]. For instance, only a fraction of interactions in online banking are manually transcribed due to costly expense. Existing semi-supervised ASR methods generate pseudo labels from an initial model trained on a small set of labeled data, which are then used to further refine the model [17]. However, these pseudo-labeling schemes are constrained by their reliance on a single 1-best hypothesis as a fixed pseudo label, limiting the model’s ability to consider other potentially correct alternatives.

**Fig 1:**
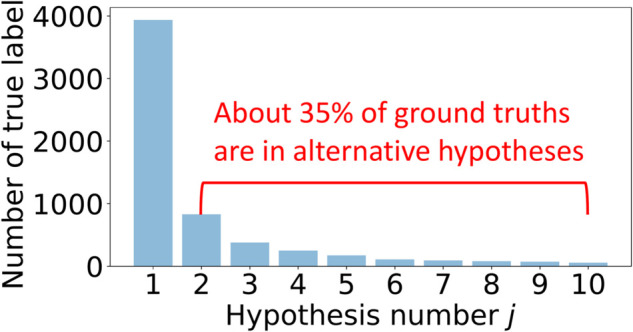
Analysis of the LJSpeech dataset showcasing how ground-truth labels are distributed across the top 10 hypotheses. Note that ground-truth labels often appear in alternative hypotheses rather than being restricted to the 1-best prediction.

To illustrate this limitation, we present the distribution of ground-truth labels in Fig 1 using the LJSpeech dataset [18]. The number *j* on the x-axis denotes the $j^{\text{th}}$-best hypothesis among the top-10 hypotheses, while the y-axis represents the counts of ground-truth label appearing in each $j^{\text{th}}$-best hypothesis. To generate these hypotheses, we use a pre-trained wav2vec 2.0 [19] finetuned on the labeled instances of LJSpeech. It is noteworthy that about 35% of ground-truth labels are overlooked in 1-best-hypothesis-based approaches. To prevent such information loss, there arises the need for approaches incorporating alternative hypotheses alongside the 1-best one.

For characterized speech, the problem of heavy reliance on inaccurate pseudo labels is exacerbated, as the scarcity of trait-specific data (e.g., Yorkshire English) often results in lower-quality pseudo labels. To supplement this scarcity, it is intuitive to incorporate unlabeled speech data from generic speakers (e.g., General American) since such data are readily available with the exponentially growing volume of speech resources today [20]. However, a key challenge lies in the significant phonetic and acoustic variation across speech traits.

**Fig 2:**
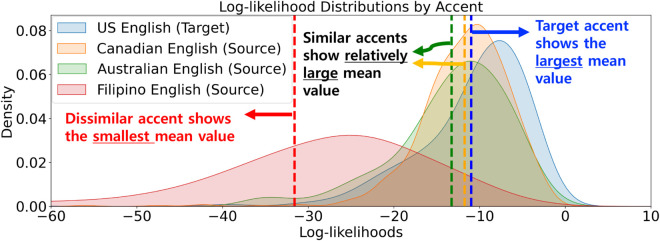
Distribution of likelihoods for the 1-best hypothesis generated by an ASR model trained on US-accented English, evaluated on speech with diverse accents. Each vertical line indicates the mean likelihood for a specific accent. The model trained on US-accented data produces higher likelihoods for phonetically similar accents (Australian and Canadian), while dissimilar accent (Filipino) results in lower likelihoods.

To further explore this challenge, we train an ASR model on US-accented English data and compare the confidence levels of predictions across various accents in Fig 2. Specifically, we compare the likelihoods of non-US-accented speech instances and those of US-accented ones. Notice that the ASR model trained on US-accented speech achieves the highest likelihoods for US-accent speech as it is optimized to maximize the likelihoods of such data. It is also noteworthy that speech with accents similar to the US accent exhibit higher likelihoods compared to those with distinct accents; Canadian and Australian accented speech yield higher likelihoods due to similar vowel sounds with US English than Filipino-accented one. This highlights that incorporating unlabeled speech data from diverse speakers without addressing phonetic and acoustic variations fails to mitigate the issue of heavy reliance on inaccurate pseudo-labels, thereby limiting model robustness.

In this work, we propose a semi-supervised learning framework to train accurate ASR models for both ordinary and characterized speech. Specifically, we propose MOCA (Multi-hypotheses-based Curriculum learning for semi-supervised Asr) for ordinary speech and MOCA-S for characterized speech. MOCA and MOCA-S incorporate multi-hypotheses-based pseudo labels for each unlabeled instance to reduce the heavy reliance on inaccurate 1-best pseudo labels. This increases the likelihood of correctly identifying the ground-truth labels and improves the robustness of the model by leveraging the diversity within multiple hypotheses. In addition, MOCA-S incorporates both unlabeled speech instances without the target trait and those with the target trait to address the scarcity of trait-specific speech data. The key idea is to adjust the influence of each unlabeled instance according to its relevance to the target trait. To further reduce the heavy reliance on the quality of the generated pseudo labels, MOCA and MOCA-S exploit curriculum learning with our novel difficulty scores. This allows the ASR model to gather more information before encountering uncertain and challenging examples, leading to more stable training; the model becomes less sensitive to the quality of pseudo-labels for challenging instances. Our contributions are summarized as follows:

- **Method.** MOCA and MOCA-S overcome the main limitation of existing methods: their heavy reliance on inaccurate 1-best pseudo labels. The key idea is to incorporate multi-hypotheses-based pseudo labels for each unlabeled instance. To minimize the model’s sensitivity to the quality of pseudo labels for difficult instances, which are often less accurate than those for easier instances, MOCA and MOCA-S exploit curriculum learning with our two novel difficulty scores.
- **Theory.** We theoretically analyze the loss function of MOCA and MOCA-S by comparing them with a traditional ASR training loss.
- **Experiments.** We conduct extensive experiments and demonstrate that MOCA and MOCA-S effectively enhance transcription performance for ordinary and characterized speech, respectively, outperforming previous methods.

The code and datasets are available at https://github.com/snudatalab/MOCA.

## Related works

In this section, we formally define the problems of ASR for ordinary and characterized speech and introduce related works.

### Problem definition

#### Semi-supervised ASR for ordinary speech.

We are given a set *X*_*L*_ of labeled speech instances and a set *X*_*U*_ of unlabeled ones with a pre-trained ASR model $f_{\theta }$, parameterized by *θ*. Then the objective of semi-supervised ASR for ordinary speech is to train an accurate transcription model $f_{\theta^{\text{new}}}$ parameterized by $\theta^{new}$ that accurately transcribes previously unseen ordinary speech instances.

#### Semi-supervised ASR for characterized speech.

We are given sets $X_{L}^{target}$ and $X_{U}^{target}$ of labeled and unlabeled speech instances with the target trait, respectively. We are also given a set $X_{U}^{non-target}$ of unlabeled speech instances with traits different from the target trait and a pre-trained ASR model $f_{\theta }$ parameterized by *θ*. Our objective is to train an accurate ASR model $f_{\theta^{new}}$ with learnable parameters $\theta^{new}$ that accurately transcribes newly given instances with the target trait.

### Pre-trained audio feature extractor for ASR

Recent advancements in speech recognition have been driven by the rise of unsupervised pre-training methods, which extract general features from speech [21–23]. These models learn from large amounts of unlabeled audio data, and their representations are directly applied in an end-to-end manner to enhance performance in downstream tasks such as speech emotion recognition [24], disease detection [25], and voice conversion [26]. The representations derived from audio properties are used to generate probable transcriptions, called hypotheses in ASR [27].

Among the commonly used pre-trained models, wav2vec [19] and wav2vec 2.0 [28] stand out for their strong performance [29,30]. These models utilize contrastive learning to distinguish similar audio pairs from dissimilar ones. The wav2vec and wav2vec 2.0 models take a speech signal $x_{i}=[x_{i;1},...,x_{i;T}]$ as input, where each $x_{i;j}\in \mathbb{R}$ corresponds to a sampled value of the speech signal at time j∈{1,2,...,T}. Their encoder produces feature representations $h_{i}=[h_{i;1},...,h_{i;T}]$, which are then mapped into a sequence of states $y_{i}=[y_{i;1},...,y_{i;T}]$ by a decoder. The decoder can be any model including an MLP. Each element $y_{i;j}\in {\mathbb{R}}^{C}$ of *y*_*i*_ represents the prediction vector at timestamp *j* for a speech input *x*_*i*_, where *C* denotes the number of output categories, including alphabet characters, blank spaces, punctuation marks, and other symbols.

### Semi-supervised ASR models

The challenge of insufficient labeled data has emerged as a critical issue across many machine learning domains [31,32], and speech is no exception. The limited availability of labeled speech data and the high costs of manual labeling have recently drawn significant attention to the development of semi-supervised ASR systems [33–36]. Previous semi-supervised ASR models employ the 1-best hypothesis as a definite pseudo label and are generally designed to accurately transcribe ordinary speech instead of characterized one. Higuchi et al. [16] employ online and offline models to enhance ASR representations through interaction. Park et al. [37] apply Noisy Student Training to ASR, introducing various levels of input augmentation. However, 1-best hypothesis approaches, which depend on a single model prediction, fail to reflect the range of possible outputs the model can generate.

Recently, there has been growing interest in developing speech models, including ASR, tailored for characterized speech [38]. These models focus on specific speech features and provide enhanced recognition accuracy in applications where general ASR systems often underperform [39,40]. Related efforts on handling characterized signals have also appeared in other domains, including wavelet-based approaches [41–43], and similar considerations have also been explored within the speech domain. Moreover, given that speech signals can be conceptually modeled as structured, high-dimensional data, insights from tensor factorization literature including spectral analysis [44,45], tensor factorization [46–48] hold promise for enhancing ASR and speech representation systems. Despite this interest, progress in semi-supervised ASR models for characterized speech remains limited. The main challenge lies in the scarcity of large volumes of both labeled and unlabeled data for specific traits [49]. To address this data scarcity, previous ASR models for characterized speech have focused on adapting to specific-featured data while leveraging general speech data [50–53]. However, these models require fully labeled data generated through a hand-crafted process, which further complicates the workflow. Note that the diversity of traits in target-featured instances makes manual transcription significantly more challenging than that of general speech, which in turn limits the model’s capabilities [54]. Kim et al. [55] propose multi-hypotheses-based pseudo labeling method for training semi-supervised ASR model, which is our preliminary work. MOCA in this paper is identical to the one proposed in our previous work [55]. In this work, we extend MOCA by additionally proposing MOCA-S, a generalized and trait-aware variant of MOCA designed for characterized speech settings. MOCA-S reduces to MOCA in the ordinary speech case, while providing extended applicability when speech traits such as accents, gender, or other characteristics are involved.

### Connectionist Temporal Classification (CTC) loss

In ASR tasks, the connectionist temporal classification (CTC) loss is widely adopted to quantify the difference between the predicted sequence of states $y_{i}=[y_{i;t}|t=1,...,T]$ and the corresponding ground-truth text label *l*_*i*_ for a given speech instance *x*_*i*_ [56]. This approach allows the model to establish an alignment between input audio frames and output characters without relying on pre-segmented data.

The CTC algorithm optimizes the likelihood of correctly predicting the ground-truth transcription *l*_*i*_ given the speech input *x*_*i*_, as formulated below:

$$p(l_{i}|x_{i})=\sum_{\pi_{i}\in {ℬ}^{-1}(l_{i})}p(\pi_{i}|x_{i}),$$ (1)

where ℬ maps *y*_*i*_ to a transcription by removing blanks and duplicate labels. Consequently, ${ℬ}^{-1}(l_{i})$ denotes the collection of all possible prediction sequences that are collapsed into the label *l*_*i*_.

The CTC loss, defined as the negative log-likelihood of p(l∣x), is given by:

$${ℒ}_{CTC}=-\sum_{i=1}^{T}\log p(l_{i}|x_{i}),$$ (2)

where *T* is the number of data instances. Note that the standard CTC loss without any additional mechanism focuses solely on the 1-best pseudo label. This limits the model’s ability to apply soft labeling across alternative labels, and makes the alignment less flexible. In contrast, our modified CTC loss enables the model to incorporate multiple hypotheses during training, allowing it to leverage a broader range of plausible alignments. This approach provides a softer, more nuanced labeling process that improves the model’s adaptability to diverse data and enhances its robustness.

## Proposed method

**Fig 3:**
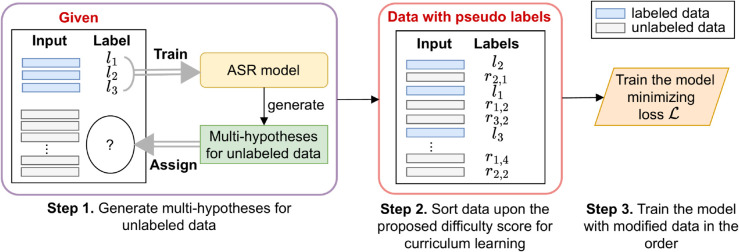
Overall framework of MOCA. We begin by training an ASR model using a small set of labeled speech samples. After training, the model produces multiple hypotheses for every unlabeled instance. For an unlabeled example *x*_*i*_, *r*_*i*,*j*_ represents the j−th pseudo label sampled from the hypothesis set *Z*_*i*_. MOCA enhances the ASR model by training with both labeled and unlabeled instances, incorporating multiple hypotheses to optimize the loss function ℒ. During training, a curriculum learning strategy incorporating a novel difficulty score is employed to mitigate over-reliance on pseudo-labels.

We propose a framework for training an accurate ASR model in a semi-supervised setting. Specifically, we propose MOCA (Multi-hypotheses-based Curriculum learning for semi-supervised Asr) for ordinary speech and MOCA-S for characterized speech. We show the overall process of MOCA and MOCA-S in Fig 3.

Initially, both MOCA and MOCA-S train an ASR model with labeled speech instances. The trained model then generates *multi-hypotheses-based* pseudo labels for each unlabeled instance. The key difference is in how instance importance is handled with pseudo-labeling: MOCA assigns a fixed number of pseudo labels to all instances equally, while MOCA-S adjusts the number of pseudo labels per instance based on its relevance to the target trait. Through this mechanism, target-relevant instances naturally receive greater weight in training, whereas less relevant instances exert a smaller influence.

**Fig 4:**
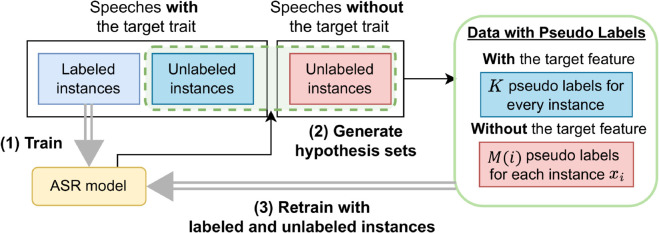
Overall framework of MOCA-S. MOCA-S initially trains an ASR model using characterized speech instances with transcription labels. Notably, MOCA-S dynamically adjusts the number of pseudo labels assigned to each unlabeled instance based on its relevance to the target feature. The ASR model is subsequently retrained by integrating both labeled and unlabeled instances with multiple hypotheses while optimizing the loss function.

Then, MOCA and MOCA-S retrain the ASR model using both labeled and unlabeled instances with pseudo labels. This process follows a defined order accounting for the difficulty and uncertainty of each example. The detailed process of MOCA-S, reflecting its key difference with MOCA, is illustrated in Fig 4. The challenges are summarized as follows:

**C1. Exclusion of ground-truth labels in pseudo labels.** For unlabeled instances, naive 1-best pseudo labels generated from a pre-trained ASR model inevitably carry uncertainties, especially when the model is trained on limited labeled instances. How can we address the uncertainties of pseudo labels?
**C2. Defining pseudo labels for unlabeled instances.** How can we train an ASR model with multiple label hypotheses for each unlabeled instance?
**C3. Disparity among traits of speech.** For training MOCA-S on characterized speech, unlabeled instances without treating distinctive traits of speech hinder the training of a trait-specific ASR model. How can we determine helpful unlabeled instances for improving the accuracy of a trait-specific ASR model?
**C4. Robustness on difficult examples.** For difficult examples, the ground-truth label may not be included in the multiple hypotheses, which leads to inaccurate pseudo labels. How can the model remain robust even for these low-accuracy instances?

We propose the following main ideas to tackle such challenges, which are discussed in detail in the following sections.

**I1. Multiple hypotheses for each unlabeled instance.** Instead of relying solely on the 1-best hypothesis, we also consider alternative hypotheses.
**I2. Sampling-based loss function.** MOCA and MOCA-S perform weighted sampling from the multi-hypotheses to generate pseudo labels for each unlabeled instance. This helps the model generate more diverse pseudo labels, allowing consideration of the uncertainties inherent in the hypotheses.
**I3. Trait-based influence adjustment of unlabeled instances.** MOCA-S reduces the influence of instances lacking the target trait while amplifying the impact of instances with the target trait during training. This is done by dynamically adjusting the number of pseudo labels for each unlabeled speech instance upon their relevance to the target trait.
**I4. Ordering instances by difficulty.** We perform curriculum learning with our novel difficulty scores. By initially training the model on easier, high-confidence instances, the model builds a strong foundation. Thus, the model becomes increasingly robust in handling challenging instances with uncertain pseudo labels as training progresses.

### Multiple hypotheses for unlabeled instances (I1)

MOCA and MOCA-S leverage a pre-trained ASR model *f*, which is trained with a small amount of labeled data, to generate pseudo-labels for unlabeled instances. One straightforward approach is to use the predicted label ${\hat{l}}_{i}$ from *f* as the pseudo-label for an unlabeled instance *x*_*i*_. However, ${\hat{l}}_{i}$ contains uncertainties, largely because the ASR model *f* is trained on a limited number of labeled examples. These uncertainties negatively affect the ASR performance.

Another approach is to use soft labels instead of the hard label ${\hat{l}}_{i}$ as commonly done in other deep learning domains [57]. This approach prevents *f* from overfitting to potentially incorrect predictions, making it more robust than the 1-best-based method. However, applying soft labeling in ASR tasks is impractical due to the overwhelming number of possible target labels. For instance, constructing *L*-letter words with the English alphabet results in 26^*L*^ possible combinations.

To efficiently manage the uncertainties associated with the pseudo labels, MOCA and MOCA-S generate a set $Z_{i}=\{z_{i;1},z_{i;2},...,z_{i;N}\}$ of *N* label hypotheses for each unlabeled speech signal *x*_*i*_, where each *z*_*i;j*_ represents a distinct label hypothesis generated for *x*_*i*_. This can be viewed as an adaptation of the soft labeling strategy tailored for ASR tasks with a constrained set of possible target labels. It provides an efficient approximation of the computationally intensive process associated with the naive soft labeling method.

We define the set *Z*_*i*_ of label hypotheses for each *x*_*i*_ as the top-*N* label candidates. These top-*N* candidates are obtained via beam search using $f_{\theta }$, which is the pre-trained ASR model trained on a small amount of labeled data and parameterized by *θ*. The probability $p(z_{i;j}|x_{i},\theta ,Z_{i})$ of selecting the *j*-th hypothesis *z*_*i;j*_ from the hypotheses set *Z*_*i*_ given a speech signal *x*_*i*_ is defined as the normalized form of beam search scores, which represent the log-likelihoods of the candidates. The sampling probability of *z*_*i;j*_ from *Z*_*i*_ is as given:

$$p(z_{i;j}|x_{i},\theta ,Z_{i})=\frac{e^{s_{i;j}}}{\sum_{k=1}^{\left|Z_{i}\right|}e^{s_{i;k}}}$$ (3)

where *s*_*i;j*_ is the beam search score for *z*_*i;j*_. Exponentiating the log-likelihood $s_{i;j}=\log p(z_{i;j}|x_{i},\theta )$ converts it into the likelihood $p(z_{i;j}|x_{i},\theta )$. Therefore, $p(z_{i;j}|x_{i},\theta ,Z_{i})$ in Eq (3) represents the normalized likelihood $p(z_{i;j}|x_{i},\theta )$ within the hypotheses set *Z*_*i*_.

### Training ASR model for ordinary speech with multiple hypotheses (I2)

Previous ASR models employ CTC loss for training, which is defined as the sum of negative log likelihoods over all speech instances. For a labeled speech instance *x*_*i*_ with its label *l*_*i*_, the likelihood $p(l_{i}|x_{i})$ is computed as in Eq (1). However, defining likelihoods for unlabeled instances is challenging as ground-truth labels are unavailable. Instead, we have multiple label hypotheses for each instance.

We define the likelihood for each unlabeled instance as the probability of observing pseudo-labels sampled from the hypothesis set *Z*_*i*_. Each element *z*_*i;j*_ is selected based on the probability $p(z_{i;j}|x_{i},\theta ,Z_{i})$ in Eq (3), where *θ* represents the parameters of the pre-trained ASR model $f_{\theta }$. This allows MOCA to generate pseudo-labels for unlabeled instances while reflecting their uncertainty levels. Sampling *K* pseudo-labels from the hypothesis set improves the model’s robustness. For example, drawing three samples from a set of 10 hypotheses provides more diverse pseudo labels compared to sampling from a set of only three hypotheses. As shown in Fig 1, a significant portion of ground-truth labels is included within the alternative hypotheses. Expanding the hypothesis set as candidates for pseudo labels enhances their selection and strengthens the model. The distribution of certainty levels among the hypotheses is also captured in the pseudo-labels, further contributing to model robustness.

Let *S*(*Z*_*i*_,*K*) represent the list of *K* pseudo-labels sampled from the hypothesis set *Z*_*i*_ for each unlabeled instance *x*_*i*_. The likelihood $p(S(Z_{i},K)|x_{i},\theta )$ of observing the sampled hypotheses *S*(*Z*_*i*_,*K*) is defined as:

$$p(S(Z_{i},K)|x_{i},\theta )=\prod_{r_{i;k}\in S(Z_{i},K)}p(r_{i;k}|x_{i},\theta )$$ (4)

We use this likelihood to represent each unlabeled instance *x*_*i*_. Consequently, MOCA minimizes the negative log-likelihood ℒ for both labeled and unlabeled instances during the retraining of the ASR model $f_{\theta^{\text{new}}}$ with updated parameters $\theta^{\text{new}}$.

$$ℒ=-\sum_{x_{i}\in X_{L}}\log p(l_{i}|x_{i},\theta^{new})-\sum_{x_{i}\in X_{U}}\sum_{r_{i;k}\in S(Z_{i},K)}\log p(r_{i;k}|x_{i},\theta^{new})$$ (5)

where *X*_*L*_ and *X*_*U*_ are sets of labeled and unlabeled speech, respectively.

### Training ASR model for characterized speech with multiple hypotheses (I3)

In the problem of semi-supervised ASR for characterized speech, we are given the three sets of speech instances: a set $X_{L}^{tar}$ of labeled speech instances with the target trait, a set $X_{U}^{tar}$ of unlabeled speech instances with the target trait, and a set $X_{U}^{non}$ of unlabeled speech instances without the target trait. We are also given a pre-trained ASR model $f_{\theta }$ parameterized by *θ*. MOCA-S first finetunes the pre-trained $f_{\theta }$ with $X_{L}^{tar}$ to build an ASR model specialized for the target trait. A naive implementation of MOCA for unlabeled instances in $X_{U}^{tar}$ and $X_{U}^{non}$ would be to construct a hypothesis set for each unlabeled instance and sample *K* hypotheses from the set to generate *K* pseudo labels. However, treating a non-target-featured instance $x_{i}^{U,non}\in X_{U}^{non}$ in the same manner as a target-featured instance $x_{j}^{U,tar}\in X_{U}^{tar}$ hinders the training of the ASR model specialized for the target trait, as $x_{i}^{U,non}$ contains traits distinct from the target trait.

To address this challenge, MOCA-S adjusts the weights of unlabeled instances in $X_{U}^{tar}$ and $X_{U}^{non}$ based on each instance’s relevance to the target trait. The main idea is to dynamically decrease the number of pseudo labels for each $x_{i}^{U,non}$ according to its relation to the target trait. This prioritizes target-featured unlabeled instances $x_{j}^{U,tar}$ in training by assigning more pseudo labels compared to $x_{i}^{U,non}$.

To measure the relation of each non-target-featured instance $x_{i}^{U,non}$ to the target trait, we employ its confidence score $s_{i;1}^{U,non}$, which is the beam search score of the 1-best hypothesis $z_{i;1}^{U,non}$ computed from the ASR model. Specifically, the number of pseudo labels for each $x_{i}^{U,non}$ is determined by the ratio of its likelihood $e^{s_{i;1}^{U,non}}$ to the mean of 1-best likelihoods of the target-featured labeled instances. Let $Z_{j}^{tar}$ and $Z_{i}^{non}$ denote the hypotheses sets for $x_{j}^{U,tar}$ and $x_{i}^{U,non}$, respectively. Then the number *M*(*i*) of pseudo labels in the sampled hypotheses set $S(Z_{i}^{non},M(i))$ for each $x_{i}^{U,non}$ is formally defined as follows:

$$M(i)=K\cdot \frac{e^{s_{i;1}^{U,non}}}{\frac{1}{\left|X_{L}^{tar}\right|}\sum_{x_{n}^{L,tar}\in X_{L}^{tar}}e^{s_{n;1}^{L,tar}}}$$ (6)

where $X_{L}^{tar}$ is the set of non-target-featured labeled instances used in the initial ASR model training and *K* is the number of pseudo labels for $x_{i}^{U,tar}\in X_{U}^{tar}$. MOCA-S employs $p(S(Z_{i}^{non},M(i))|x_{i}^{U,non},\theta )$ as the likelihood for each $x_{i}^{U,non}$ following MOCA. MOCA-S minimizes the negative log likelihood for both labeled and unlabeled instances during the retraining of ASR model $f_{\theta^{\text{new}}}$ with a new parameter $\theta^{\text{new}}$. The loss function ${ℒ}_{S}$ of MOCA-S for retraining $f_{\theta^{\text{new}}}$ is expressed as follows:

$${ℒ}_{S}=-\sum_{x_{n}^{L,tar}\in X_{L}^{tar}}\log p(l_{n}|x_{n}^{L,tar},\theta^{new})\;-\sum_{x_{j}^{U,tar}\in X_{U}^{tar}}\sum_{r_{j;k}^{tar}\in S(Z_{j}^{tar},K)}\log p(r_{j;k}^{tar}|x_{j}^{U,tar},\theta^{new})\;-\sum_{x_{i}^{U,non}\in X_{U}^{non}}\sum_{r_{i;m}^{non}\in S(Z_{i}^{non},M(i))}\log p(r_{i;m}^{non}|x_{i}^{U,non},\theta^{new})$$ (7)

where $r_{h;k}^{tar}$ and $r_{h;k}^{non}$ are the k−th pseudo labels sampled from the hypotheses sets for each unlabeled example $x_{h}^{U,tar}$ and $x_{h}^{U,non}$, respectively. $X_{L}^{tar}$ contains target-featured labeled instances; $X_{U}^{tar}$ and $X_{U}^{non}$ consist of unlabeled instances with and without the target trait, respectively. This dynamic sampling in MOCA-S adjusts the number of pseudo labels for non-target-featured unlabeled instances, enabling better control over the relative impact of target-featured and non-target-featured unlabeled instances in the loss calculation.

### Curriculum learning (I4)

If all pseudo-labels within the sampled hypothesis set *S* for an unlabeled instance *x*_*i*_ are incorrect, relying on multiple pseudo-labels still inherits the uncertainty of the initial ASR model. To mitigate excessive dependence on pseudo-label quality, MOCA and MOCA-S introduce a curriculum learning strategy that incorporates a novel difficulty scoring mechanism for each speech instance. The core principle is to prioritize training on easier examples, which exhibit higher certainty, before gradually incorporating more challenging and uncertain ones. This progressive approach helps the model effectively learn complex decision boundaries.

We introduce two different difficulty scoring methods for curriculum learning. The first method considers a speech instance as more difficult if it is spoken at a faster rate. This is motivated by real-world situations, where speakers often slow down when communicating with young children, elderly listeners, or when repeating themselves to ensure better comprehension. The observation [58] that higher speech rates yield higher ASR error rates supports such motivation. The scoring method also considers longer transcriptions more difficult, as longer label sequences tend to yield higher word error rates [59]. The score is defined as:

$$difficulty\_score(x_{i})=\frac{len(l_{i})}{len(x_{i})}*len(l_{i})$$ (8)

where *l*_*i*_ represents a (pseudo) label for a speech instance *x*_*i*_, and len(·) denotes a function returning the length of its input. This score is derived by multiplying the speech rate, $len(l_{i})/len(x_{i})$, with the length of the uttered sentence, $len(l_{i})$.

The second method, similar to the first, also considers longer sentences more challenging. However, it further differentiates instances of the same length based on prediction confidence, treating those with higher confidence as easier. This design naturally reflects real-world factors, since model confidence tends to decrease for speech that differs acoustically or contextually from the target domain. For instance, utterances recorded in noisy environments or with strong emotional expression are often recognized with lower confidence, making them effectively harder examples in the curriculum. Using the model’s posterior confidence adds a model-centric view of difficulty, which has been shown to improve curriculum learning in end-to-end ASR systems [60]. The corresponding difficulty score is given by:

$$difficulty\_score(x_{i})=len(l_{i})+(1-p(l_{i}|x_{i}))$$ (9)

For all instances including unlabeled ones, difficulty scores are computed, and the ASR model is trained starting with lower-scoring examples. The appropriate difficulty score for each dataset is selected based on experimental results.

### Theoretical analysis

MOCA and MOCA-S use the sampling-based loss ℒ and ${ℒ}_{S}$ in Eqs (5) and (7), respectively, to optimize the parameters $\theta^{\text{new}}$ of the ASR model $f_{\theta^{\text{new}}}$. We theoretically analyze the relationship between the loss functions (ℒ and ${ℒ}_{S}$) and the CTC loss.

**Theorem 1.** (Relationship between ℒ of MOCA and CTC Loss) *Let* $z_{i}\in Z_{i}$ *denote a latent variable representing the pseudo label of an unlabeled instance x*_*i*_ *where Z*_*i*_ *is the set of label hypotheses for x*_*i*_, $\theta^{new}$ *be the model parameter, and S*(*Z*_*i*_,*K*) *be the list of K-sampled pseudo labels from Z*_*i*_*. Then the loss* $ℒ(x_{i})$ *of MOCA for each unlabeled instance* $x_{i}\in X_{U}$ *is the expectation of CTC loss in terms of z*_*i*_ *with a balancing factor* |*S*(*Z*_*i*_,*K*)|:

$$ℒ(x_{i})=-\sum_{r_{i;k}\in S(Z_{i},K)}\log p(r_{i;k}|x_{i},\theta^{new})\;\approx \left|S(Z_{i},K)\right|\cdot {𝔼}_{z_{i}\sim p(z_{i}|x_{i},\theta ,Z_{i})}[-\log p(z_{i}|x_{i},\theta^{new})]$$ (10)

*Proof*: MOCA generates the set *S*(*Z*_*i*_,*K*) of pseudo labels for each unlabeled instance *x*_*i*_ by sampling *K* examples from *Z*_*i*_. The loss term $ℒ(x_{i})$ of each unlabeled instance $x_{i}\in X_{U}$ in Eq (10) is rewritten as follows:

$$ℒ(x_{i})=-\sum_{z_{i}\in Z_{i}}\#z_{i}\cdot \log p(z_{i}|x_{i},\theta^{new})$$ (11)

where $\#z_{i}$ is the number of *z*_*i*_ in *S*(*Z*_*i*_,*K*). Since $z_{i}\in Z_{i}$ follows the distribution $p(z_{i}|x_{i},\theta ,Z_{i})$ in Eq (3), $\frac{\#\;of\;z_{i}}{\left|S(Z_{i},K))\right|}$ is the empirical probability of $p(z_{i}|x_{i},\theta ,Z_{i})$. Then $ℒ(x_{i})$ in Eq (7) is expressed as follows:

$$ℒ(x_{i})=-|S(Z_{i},K)|\sum_{z_{i}\in Z_{i}}\frac{\#z_{i}}{\left|S(Z_{i},K)\right|}\cdot \log p(z_{i}|x_{i},\theta^{new})\;\approx -|S(Z_{i},K)|\sum_{z_{i}\in Z_{i}}p(z_{i}|x_{i},\theta ,Z_{i})\log p(z_{i}|x_{i},\theta^{new})\;=|S(Z_{i},K)|\cdot {𝔼}_{z_{i}\sim p(z_{i}|x_{i},\theta ,Z_{i})}[-\log p(z_{i}|x_{i},\theta^{new})]$$ (12)

which ends the proof. □

**Theorem 2.** (Relationship between ${ℒ}_{S}$ of MOCA-S and CTC Loss) *Let* $z_{i}^{U,non}\in Z_{i}^{non}$ *represent a latent variable corresponding to the pseudo label of a non-target-featured unlabeled instance* $x_{i}^{U,non}\in X_{U}^{non}$*, where* $Z_{i}^{non}$ *denotes the set of label hypotheses for* $x_{i}^{U,non}$, $\theta^{new}$ *be the model parameter, and* $S(Z_{i}^{non},M(i))$ *be the list of M(i)-sampled pseudo labels from* $Z_{i}^{non}$. *The loss term* ${ℒ}_{S}(x_{i}^{U,non})$ *of MOCA-S for each non-target-featured unlabeled instance* $x_{i}^{U,non}$ *is the expectation of the CTC loss over* $z_{i}^{U,non}$*, scaled by the balancing factor* $\left|S(Z_{i}^{non},M(i))\right|$:

$${ℒ}_{S}(x_{i}^{U,non})=-\sum_{r_{i;m}^{non}\in S(Z_{i}^{non},M(i))}\log p(r_{i;m}^{non}|x_{i}^{U,non},\theta^{new})\;\approx |S(Z_{i}^{non},M(i))|\cdot {𝔼}_{z_{i}^{U,non}\sim p(z_{i}^{U,non}|x_{i}^{U,non},\theta ,Z_{i}^{non})}[-\log p(z_{i}^{U,non}|x_{i}^{U,non},\theta^{new})]$$ (13)

*Proof*: MOCA-S constructs the set $S(Z_{i}^{non},M(i))$ of *M*(*i*)-sampled pseudo labels for each non-target-featured unlabeled instance $x_{i}^{U,non}\in X_{U}^{non}$. The loss term ${ℒ}_{S}(x_{i}^{U,non})$ for non-target-featured instances in Eq (13) is expressed as follows:

$${ℒ}_{S}(x_{i}^{U,non})=-\sum_{z_{i}^{U,non}\in Z_{i}^{non}}\#z_{i}^{U,non}\cdot \log p(z_{i}^{U,non}|x_{i}^{U,non},\theta^{new})$$ (14)

where $\#z_{i}^{U,non}$ is the count of $z_{i}^{U,non}$ in $S(Z_{i}^{non},M(i))$. The distribution $p(z_{i}^{U,non}|x_{i}^{U,non},\theta ,Z_{i}^{non})$ for $z_{i}^{U,non}\in Z_{i}^{non}$ follows Eq (3) and $\frac{\#z_{i}^{U,non}}{\left|S(Z_{i}^{non},M(i))\right|}$ is the empirical probability of $p(z_{i}^{U,non}|x_{i}^{U,non},\theta ,Z_{i}^{non})$. Reformulating ${ℒ}_{S}(x_{i}^{U,non})$ in Eq (13) yields the following expression:

$$L_{S}(x_{i}^{U,non})=-\left|S(Z_{i}^{non},M(i))\right|\sum_{z_{i}^{U,non}\in Z_{i}^{non}}\frac{\#z_{i}^{U,non}}{\left|S(Z_{i}^{non},M(i))\right|}\cdot \log p(z_{i}^{U,non}|x_{i}^{U,non},\theta^{new})\;\approx -\left|S(Z_{i}^{non},M(i))\right|\sum_{z_{i}^{U,non}\in Z_{i}^{non}}p(z_{i}^{U,non}|x_{i}^{U,non},\theta ,Z_{i}^{non})\log p(z_{i}^{U,non}|x_{i}^{U,non},\theta^{new})\;=\left|S(Z_{i}^{non},M(i))\right|\cdot {𝔼}_{z_{i}^{U,non}\sim p(z_{i}^{U,non}|x_{i}^{U,non},\theta ,Z_{i}^{non})}[-\log p(z_{i}^{U,non}|x_{i}^{U,non},\theta^{new})]\;$$ (15)

hence the proof. □

## Experiments

To explore the following key research questions, we carry out experiments across four real-world datasets, each with distinct settings.

**Q1. Transcription Performance for Ordinary Speech.** How accurately does MOCA transcribe speech from general population into texts compared to the baseline models in a semi-supervised setting?
**Q2. Transcription Performance for Characterized Speech.** How accurately does MOCA-S transcribe speech instances from speakers with a specific trait compared to the baselines in a semi-supervised setting?
**Q3. Training Trend under Multi-hypotheses.** How does the number of hypotheses per unlabeled instance affect the training trajectory?
**Q4. Ablation Study.** Does each module of MOCA and MOCA-S improve transcription performance?

### Experimental settings

We present the experimental settings, including datasets, baselines, and evaluation metrics. All experiments are conducted on a single GPU machine with a GTX 3080.

#### Dataset.

We use four real-world speech datasets to evaluate MOCA and MOCA-S. The data statistics are summarized in Table 1. We evaluate MOCA on the LJSpeech [18] and LibriSpeech-dev-clean [61] datasets, which contain recordings from a general population. LJSpeech dataset comprises 13,100 audio clips, totaling approximately 24 hours of clear English speech from a single female speaker. LJSpeech includes passages from seven non-fiction books, with each clip accompanied by a transcription as its label. LibriSpeech-dev-clean consists of 2,360 speech signals sourced mainly from the LibriVox project audiobooks, amounting to approximately 5.4 hours. In our experiments, we used dev-clean and test-clean for independent training to emulate a data-scarce environment, aiming to demonstrate the effectiveness of MOCA in semi-supervised learning scenarios. For MOCA-S, we evaluate the performance using CommonVoice [62] and SLR83 [63], which contain recordings from speakers with various characteristics. CommonVoice [62] includes 18,374 audio clips with specific traits of speakers such as age, gender, and accent. SLR83 [63] contains 10,627 audio speech instances of speakers from six distinct regions of England and Ireland.

**Table 1:** Summary of datasets.

Dataset	Hours	Number of speakers	Language
LJSpeech^1^	24.0	1	English
LibriSpeech-dev/test-clean^2^	5.4	40	English
CommonVoice^3^	33	617	English
SLR83^4^	18.2	71	English

^1^ https://keithito.com/LJ-Speech-Dataset/

^2^ http://www.openslr.org/12

^3^ https://commonvoice.mozilla.org/en/datasets

^4^ https://openslr.trmal.net/resources/83/about.html

#### Baselines.

We compare MOCA and MOCA-S with previous ASR methods. Supervised [19] is a basic ASR model that loads pre-trained wav2vec 2.0 and fine-tunes it on labeled speech data. 1-best utilizes the trained Supervised model to generate 1-best pseudo labels for unlabeled speech instances. Self-train [64] is a self-training-based semi-supervised ASR method that dynamically generates pseudo labels during training. Both 1-best and Self-train build on the Supervised backbone and leverage both labeled and pseudo-labeled data. The key difference is that 1-best relies on fixed pseudo labels generated once at the beginning, whereas Self-train continually regenerates them with the updated model during training.

#### Evaluation metrics.

We use Word Error Rate (WER) and Character Error Rate (CER) as evaluation metrics to assess the performance of MOCA and MOCA-S. These metrics are commonly used in speech recognition to quantify transcription accuracy. The WER metric is defined as follows:

$$\text{WER}=\frac{1}{T}\sum_{t=1}^{T}\frac{S_{w}^{t}+D_{w}^{t}+I_{w}^{t}}{N_{w}^{t}}$$ (16)

where $S_{w}^{t}$, $D_{w}^{t}$, and $I_{w}^{t}$ are the numbers of word-level substitutions, deletions, and insertions in *t*-th data instance, respectively. $N_{w}^{t}$ is the total number of words in the true transcription of the *t*-th data instance, and *T* is the total number of data instances. Similarly, the CER metric is calculated by:

$$\text{CER}=\frac{1}{T}\sum_{t=1}^{T}\frac{S_{c}^{t}+D_{c}^{t}+I_{c}^{t}}{N_{c}^{t}}$$ (17)

where $S_{c}^{t}$, $D_{c}^{t}$, and $I_{c}^{t}$ denote the number of character-level substitutions, deletions, and insertions for the *t*-th instance, respectively, and $N_{c}^{t}$ is the total number of characters in the ground-truth transcription for the *t*-th instance.

#### Settings for MOCA.

The LJSpeech and LibriSpeech datasets are divided into training-labeled, training-unlabeled, and test sets with ratios of 1:4:5 and 5:4:1, respectively, taking into account the limited number of speech samples in LibriSpeech. Specifically, we apply the 5:4:1 split independently to the dev-clean and test-clean subsets of LibriSpeech, using each subset in its entirety to construct separate training and evaluation sets. The training-labeled set consists of speech instances paired with transcription labels, while the training-unlabeled set comprises instances lacking transcriptions. The hypothesis pool is set to include 10 candidates, and the number *K* of sampled pseudo label is selected from {3,5,10}. Wav2vec 2.0 is adopted as the base ASR model, with its initial parameters configured as described in [21]. We train the initial ASR model on labeled instances for 100 epochs, followed by 60 epochs of fine-tuning on all instances, including labeled and pseudo-labeled ones.

#### Settings for MOCA-S.

For CommonVoice and SLR83, we set Northern Irish and Southern English accent as the target trait, respectively. We split instances of the target trait into training-labeled, training-unlabeled, and test sets with ratio of 0.8:0.1:0.1 for CommonVoice and 0.6:0.3:0.1 for SLR83. Note that all speech without the target trait are included in the training-unlabeled set; only a subset of speech with the target trait are labeled. We set the hypotheses pool to include 10 candidates and vary the maximum number *K* of sampled pseudo labels in Eq (6) among {3,5,10}. We employ wav2vec 2.0 as our base ASR model, initializing parameters as specified in [21]. We train the initial model for 150 epochs, followed by 20 epochs of fine-tuning.

### Transcription performance of MOCA for ordinary speech (Q1)

We evaluate the transcription performance of MOCA against the baselines, as shown in Table 2. MOCA significantly reduces both WER and CER compared to the 1-best model. Notably, using the number *K* = 5 of sampled pseudo labels yields better transcription accuracy than *K* = 3, highlighting the benefit of incorporating more pseudo labels from the hypothesis pool. However, an overly large *K* introduces excessive variability, negatively impacting the performance.

**Table 2:** ASR performance of MOCA and baselines measured by WER and CER. Bold and underlined numbers represent the best and second-best results, respectively. MOCA outperforms the competitors across various settings.

Model	# of sampled hypotheses *K*	Difficulty score	LJSpeech		dev-clean		test-clean	
			WER	CER	WER	CER	WER	CER
Supervised	N/A	N/A	10.08	2.23	21.60	6.75	19.68	6.19
1-best	N/A	N/A	8.21	1.74	16.22	5.28	15.20	4.30
Self-train	N/A	N/A	8.99	2.01	16.16	5.34	17.16	5.07
MOCA	3	confidence	6.85	1.44	15.23	4.80	15.11	4.19
	3	speed	6.88	1.48	15.32	4.94	**15.01**	4.22
	5	confidence	**6.76**	**1.43**	15.69	4.87	15.05	**4.10**
	5	speed	6.98	1.51	15.21	**4.72**	15.32	4.48
	10	confidence	6.85	1.47	15.86	**4.72**	15.24	4.23
	10	speed	8.87	2.14	**14.83**	4.76	15.75	4.63

### Transcription performance of MOCA-S for characterized speech (Q2)

We evaluate transcription performance of MOCA-S for the target-featured speech and the baselines in Table 3. WER and CER of MOCA-S improve compared to those of 1-best and self-train models. Sampling more pseudo labels for target-featured speech instances represents higher transcription accuracy. As MOCA-S lacks in quantity of target-featured speech instances, effectively employing more instances considering the relation to the target trait improves the transcription performance, relieving such data scarcity problem.

**Table 3:** ASR performance of MOCA-S and baselines measured by WER and CER. Bold and underlined numbers represent the best and second-best results, respectively. MOCA-S outperforms the competitors across various settings.

Model	# of sampled hypotheses K	Difficulty score	CommonVoice		SLR83	
			WER	CER	WER	CER
Supervised	N/A	N/A	17.27	4.31	14.21	4.54
1-best	N/A	N/A	15.34	3.61	13.85	4.33
Self-train	N/A	N/A	16.30	3.87	14.11	4.30
MOCA-S	3	confidence	16.43	3.85	13.65	4.34
	3	speed	15.27	3.51	13.97	4.30
	5	confidence	16.91	3.89	13.54	4.31
	5	speed	**14.60**	**3.41**	13.96	4.33
	10	confidence	15.92	3.80	13.83	4.34
	10	speed	15.07	3.43	13.33	**4.21**

### Training trend under multi-hypotheses (Q3)

**Fig 5:**
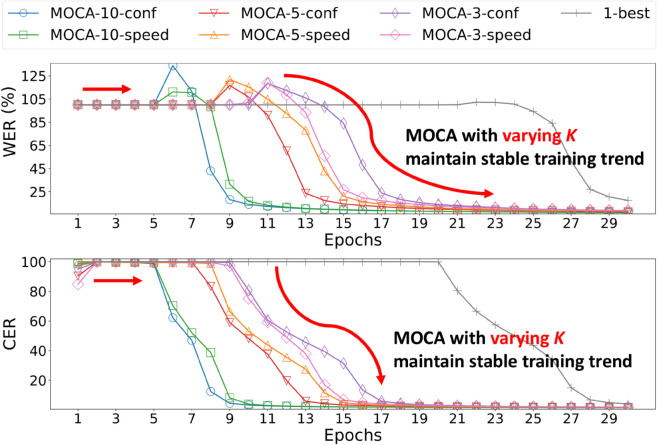
Transcription performance of MOCA over epochs on the LJSpeech dataset. MOCA shows stable convergence behavior across varying *K*. This confirms the robustness of MOCA in maintaining stable learning dynamics under varying degrees of pseudo-label uncertainty.

We analyze the training behavior of MOCA with varying numbers *K* of hypotheses per unlabeled utterance in Fig 5. We evaluate two curricula, MOCA-*K*-conf and MOCA-*K*-speed, which use confidence-based and speed-based difficulty scores, respectively, with *K* sampled pseudo labels. The number of updates per epoch increases with *K*, since each unlabeled utterance is associated with *K* pseudo labels. Each epoch denotes one sweep over labeled data and pseudo labels per unlabeled instance.

Although a larger *K* increases pseudo-label diversity and may lead to performance fluctuations or divergent learning behaviors due to noisy labels, all models are observed to converge in a consistent and stable manner. For instance, MOCA-10-conf exhibits a more pronounced increase in error during the early stages, presumably due to the higher likelihood of incorporating incorrect pseudo labels. Nevertheless, it eventually converges stably without further degradation. In contrast, models with smaller *K* show a more stable start, followed by a similar convergence trend overall. This stability is particularly desirable in semi-supervised training. These results demonstrate that MOCA maintains robust learning dynamics even under high pseudo-label uncertainty, effectively mitigating the impact of noise.

### Ablation study (Q4)

We analyze the contribution of each module in MOCA and MOCA-S through the following ablation variants, summarized in Tables 4 and 5:

**Table 4:** Ablation study for MOCA. The best performance is highlighted in bold. Each module plays a crucial role in enhancing the overall transcription performance.

Model	dev-clean		test-clean	
	WER	CER	WER	CER
MOCA-1-best	15.76	4.93	16.83	5.19
MOCA-uniform-sampling	16.20	4.84	23.19	6.60
MOCA-w/o-curriculum	15.29	4.98	16.08	**4.59**
MOCA-inverse-curriculum	18.72	5.81	21.67	6.36
MOCA	**14.83**	**4.76**	**15.75**	4.63

**Table 5:** Ablation study for MOCA-S. The best performance is highlighted in bold. Each module plays a crucial role in enhancing the overall transcription performance.

Model	CommonVoice		SLR83	
	WER	CER	WER	CER
MOCA-S-1-best	14.79	3.44	13.76	4.41
MOCA-S-uniform-sampling	17.00	3.93	14.22	4.38
MOCA-S-w/o-curriculum	15.06	3.56	14.13	4.38
MOCA-S-inverse-curriculum	17.14	4.07	14.13	4.46
MOCA-S-target-curriculum	17.21	3.97	13.73	4.33
MOCA-S-fixed-K	17.15	4.09	14.22	4.44
MOCA-S	**14.60**	**3.41**	**13.33**	**4.21**

- MOCA/MOCA-S-1-best: uses only the top-1 pseudo label to examine the effect of leveraging multiple hypotheses versus a single one.
- MOCA/MOCA-S-uniform-sampling: samples pseudo labels with equal weights rather than by model likelihoods to test the effect of likelihood-based weighting.
- MOCA/MOCA-S-w/o-curriculum: removes curriculum learning to test the role of progressive difficulty scheduling.
- MOCA/MOCA-S-inverse-curriculum: trains from the hardest to the easiest instances, used to evaluate the effect of reversing the proposed easy-to-hard order.
- MOCA-S-target-curriculum: prioritizes speech with the target feature before non-target-featured instances to examine whether explicit trait-based ordering provides additional benefit beyond multiple hypothesis generation.
- MOCA-S-fixed-K: uniformly samples a fixed number *K* of pseudo labels for non-target-featured unlabeled instances to assess the importance of dynamically adjusting *M*(*i*) according to trait relevance.

For MOCA, we set the number *K* of sampled hypotheses to 10 and use speed-based difficulty scores for curriculum learning. For MOCA-S, the same difficulty scores are used with *K* = 5 for CommonVoice and *K* = 10 for SLR83.

Tables 4 and 5 show that each module of MOCA and MOCA-S contributes meaningfully to transcription accuracy. Both MOCA and MOCA-S outperform the 1-best and uniform-sampling variants, confirming the benefit of weighting pseudo labels by model likelihoods. They also surpass the w/o-curriculum and inverse-curriculum variants, with inverse-curriculum yielding the lowest performance, underscoring the value of the proposed curriculum design.

In Table 5, MOCA-S achieves lower error rates than MOCA-S-target-curriculum, which performs the worst on CommonVoice. Because multiple hypothesis generation already addresses the disparity between target-featured and non-target-featured speeches, enforcing a fixed trait-based order harms performance. MOCA-S also outperforms MOCA-S-fixed-K, indicating that sampling a fixed number of pseudo labels without considering trait relevance reduces effectiveness. Overall, these ablation results demonstrate the necessity of both dynamic sampling and curriculum learning for robust semi-supervised training.

## Supplementary experiments

### Additional results on gender-based speech

To further validate the applicability of MOCA-S beyond accent-based domains, we conduct an additional experiment using the SLR83 dataset. While our main experiments treat accents as the defining characterization, we reinterpret SLR83 by splitting speech based on gender (male vs. female), thereby creating a different type of domain variation. Table 6 reports the results when targeting male speech. The baseline methods Supervised, 1-best, and Self-train yield WERs of 14.25, 13.90, and 14.08, respectively. In contrast, MOCA-S achieves a WER of 13.75, obtaining lower error rates than the baselines. A similar trend is observed for CER: Supervised, 1-best, and Self-train achieve CERs of 4.41, 4.34, and 4.38, respectively, while MOCA-S attains 4.33. These results demonstrate that MOCA-S is not restricted to accent-based characterization. MOCA-S effectively captures domain variations beyond accents, such as gender differences, thereby broadening its applicability to a wider range of characterized speech scenarios.

**Table 6:** Results on SLR83 when targeting male speech. The best performance is highlighted in bold. MOCA-S consistently outperforms baselines.

	Supervised	1-best	Self-train	MOCA-S-10-speed
WER	14.25	13.90	14.08	**13.75**
CER	4.41	4.34	4.38	**4.33**

### Robustness of MOCA and MOCA-S

We evaluate MOCA on LJSpeech and MOCA-S on SLR83 to examine its robustness. All experiments are conducted five times with different random seeds, and the reported results correspond to the mean and standard deviation. We use *K* = 5 with confidence-based difficulty score for MOCA and *K* = 10 with speed-based difficulty score for MOCA-S following the best performed model configuration in Tables 2 and 3, respectively. As shown in Tables 7 and 8, both MOCA and MOCA-S consistently outperform the baseline methods in terms of WER and CER. Furthermore, the relatively small deviations across runs in MOCA indicate that the improvements are stable and not sensitive to random initialization, demonstrating its robustness.

**Table 7:** Results on LJSpeech. The best performance is highlighted in bold. MOCA consistently outperforms baselines.

	Supervised	1-best	Self-train	MOCA-5-conf
WER	9.44 ± 0.91	8.79 ± 0.82	8.44 ± 0.78	**6.83 ± 0.09**
CER	2.07 ± 0.23	1.87 ± 0.18	1.86 ± 0.22	**1.44 ± 0.01**

**Table 8:** Results on SLR83. The best performance is highlighted in bold. MOCA-S consistently outperforms baselines.

	Supervised	1-best	Self-train	MOCA-S-10-speed
WER	14.22 ± 0.01	13.84 ± 0.02	14.17 ± 0.08	**13.60 ± 0.38**
CER	4.49 ± 0.07	4.34 ± 0.01	4.35 ± 0.06	**4.27 ± 0.08**

## Discussion

This work demonstrates that multi-hypothesis pseudo labeling combined with curriculum learning significantly enhances semi-supervised ASR for both ordinary and characterized speeches, directly tackling the challenge of limited labeled data. By sampling multiple hypotheses and guiding training through difficulty scores, MOCA and MOCA-S capture richer information than conventional 1-best methods [33,34,36]. This avoids over-reliance on a single prediction and better reflects the range of potential ground-truth labels. Interestingly, although we expected explicit target-prioritized ordering to improve the performance of MOCA-S, it did not. This outcome indicates that additional heuristics may even hinder training, and that our multi-hypothesis design already manages trait disparities effectively. While we did not extend our experiments to low-resource or multilingual ASR, this boundary of scope highlights that such scenarios require original ideas to properly address cross-lingual variation. We regard this as an exciting avenue for future research. In this sense, our framework provides a strong foundation that not only advances semi-supervised ASR but also inspires broader extensions to increasingly diverse speech scenarios.

### Future works

We outline how our multi-hypotheses-based curriculum-learning framework generalizes to diverse ASR settings.

#### Low-resource ASR.

Low-resource ASR corresponds to scenarios where only a small amount of transcribed speech is available, ranging typically just a few hours. In such cases, models must rely heavily on self-supervised representations and pseudo-labeling strategies. MOCA is well-suited to this context, as MOCA is designed to operate effectively with minimal supervision. The training process of MOCA is directly applicable by generating multiple hypotheses using a pretrained model (e.g., wav2vec 2.0 [28]) with beam decoding, followed by instance-level sampling and curriculum-based scheduling. Modification to the underlying architecture or loss function is not required, making MOCA a plug-in strategy for improving learning efficiency in data-scarce environments.

#### Multilingual ASR.

Multilingual ASR aims to build a unified model capable of transcribing speech from multiple languages. This setting introduces challenges such as language identification, cross-lingual generalization, and imbalanced data distributions across languages. While our current work does not target this setting directly, the core ideas behind MOCA-S can be extended to multilingual contexts. For example, each language can be treated as a distinct domain, with the number and training order of pseudo labels adjusted using cross-lingual similarity metrics (e.g., phonetic or embedding-based distance). However, directly realizing such an extension is difficult since substantial differences in word order, phonetic inventory, and vocabulary pools require not only technical adjustments but also novel ideas for modeling language-level uncertainty and adaptation. We therefore leave this as an important direction for future work.

To summarize, our framework is readily applicable to low-resource ASR settings. While additional mechanisms to account for language-specific variation and domain-level uncertainty are required, extensions to multilingual ASR are conceivable.

## Conclusions

In this study, we propose MOCA and MOCA-S, robust semi-supervised ASR methods for ordinary and characterized speech, respectively. MOCA and MOCA-S address the critical limitations of existing pseudo-labeling based approaches, particularly in handling pseudo-label uncertainty. The main idea is to incorporate multi-hypotheses-based pseudo labels for the unlabeled instances. MOCA-S dynamically adjusts the number of pseudo labels for non-target-featured speech instances based on the target trait. This ensures that non-target-featured data meaningfully enriches training, effectively addressing data scarcity challenges of target-featured instances. Additionally, our curriculum learning strategy with a tailored difficulty score prioritizes easier examples in initial training phases, allowing the model to progressively tackle more complex cases. This structured learning process minimizes reliance on pseudo-label quality and improves the model’s robustness. Experimental results on real-world datasets demonstrate improved transcription performance and faster convergence, underscoring the efficiency of our multi-hypotheses and adaptive sampling techniques in building robust ASR models for both ordinary and characterized speeches.
